# Wildlife Disease Monitoring: Methods and Perspectives

**DOI:** 10.3390/ani12213032

**Published:** 2022-11-04

**Authors:** Maria V. Mazzamuto, Anna-Katarina Schilling, Claudia Romeo

**Affiliations:** 1 Haub School of Environment and Natural Resources, University of Wyoming, 1000 E. University Ave, Laramie, WY 82072, USA; 2 Department of Theoretical and Applied Sciences, University of Insubria, Via J.H. Dunant 3, 21100 Varese, Italy; 3 Previously Royal (Dick) School of Veterinary Studies and Roslin Institute, University of Edinburgh, Easter Bush, Midlothian EH25 9RG, UK; 4 Istituto Zooprofilattico Sperimentale della Lombardia e dell’Emilia Romagna (IZSLER), Via Bianchi 9, 25124 Brescia, Italy

In the last few decades, scientific interest in wildlife diseases has steadily grown and has recently been boosted by the SARS-CoV-2 pandemic, which highlighted that the health of humans, livestock, wildlife and, ultimately, of the whole environment is inextricably linked. Within such a One Health framework, wildlife disease monitoring and surveillance have become paramount from a public health perspective. Additionally, anthropogenic changes—e.g., climate change, growing urbanization, biological invasions—increasingly threaten biodiversity and appear to have favoured disease emergence events in wild animals, making wildlife disease research highly relevant from a conservation perspective. There is therefore a growing need for a better understanding of disease circulation in natural populations. Despite the many recent advances in epidemiology and diagnostic methods, wildlife disease research remains a challenging field due to the intrinsic difficulties related to the logistics of field sampling, the necessity for non-invasive methods, and the frequent lack of validated analytical tests or prior data on specific diseases in a species or population. The eleven articles and two reviews included in the Special Issue entitled “Wildlife Disease Monitoring: Methods and Perspectives” are thus focused on methodologies and approaches that may facilitate wildlife disease research and help overcome its many intrinsic issues, either in a public health, ecological research or conservation context.

The World Organisation for Animal Health (WOAH, formerly OIE) recommends the implementation of national, coordinated wildlife health surveillance (WHS) programmes aimed at effectively managing issues related to pathogens in wildlife and inform disease management to better safeguard human and animal health. In their contribution, Lawson et al. [1] collate the experiences, points of view and solutions that emerged during a dedicated workshop organised by the European Wildlife Disease Association and attended by a broad audience of experienced researchers, stakeholders and representatives from several European countries. The authors first describe the set up and growth of successful national WHS programmes in Europe, then list the main challenges to WHS implementation that emerged during the workshop and finally offer useful recommendations on how to tackle them.

Several contributions within this Special Issue report on the methodology and results of long-term, structured disease surveillance and/or health monitoring programs, either general or targeted toward specific pathogens or animal species. Maaz et al. [2] offer a best-practice example of a large-scale standardised sampling approach for game animals employed by the German Federal Institute for Risk Assessment (BfR) for the detection of both biological and chemical agents in wildlife. They present the detailed methodology and output of their sampling protocol, emphasizing the involvement of local officers and hunters as a key element for its correct implementation. While acknowledging the limitations and potential biases of such an approach, the authors highlight the benefits of the long-term acquisition of samples, coupled with the efficient use of manpower and time.

Vengušt et al. [3] present valuable, large-scale disease data on chamois (*Rupicapra rupicapra*) populations in Slovenia gathered through passive surveillance. In their paper, they describe the main pathogen-associated findings on chamois carcasses retrieved in the country over a 20-year time span, identifying parasitic diseases, and, in particular, sarcoptic mange, as the main cause of mortality in this alpine ungulate. A focus on mange as a driver of chamois population dynamics in the Alps is provided by Obber et al. [4], who summarise the epidemiological and demographic data collected over the last 15 years within a local mange monitoring strategy in Trento province (Italy). The authors further discuss the advantages of setting up a passive surveillance programme to monitor the evolution of disease outbreaks and evaluate the costs and benefits of employing an enhanced surveillance approach based on periodic intensive censuses. 

Two surveys add valuable knowledge on bacterial infections in a European mammal of conservation concern: the European bison (*Bison bonasus*). Both studies were carried out as part of a health monitoring program within a national bison conservation project in Poland, where the largest surviving population of the species lives. Kwiecień et al. [5] present data on infection with *Trueperella pyogenes* and its genetic diversity collected from a large sample of Polish bison over 10 years. Their survey reveals a worrisome prevalence of the infection (almost 15%), and the authors suggest a role of the bacteria in the pathogenesis of *balanoposthitis*, a chronic disease that may lead to reproduction disorders and that has been recognised in bison since the 1980s. Following bovine tuberculosis outbreaks in bison inhabiting the region, Didkowska et al. [6] investigated whether *Mycobacterium avium* spp. *paratuberculosis* (MAP), for which wild and domestic ruminants are normally reservoirs, represented a potential additional threat to the species. Based on their findings, the authors exclude a major role for MAP as a threat to bison and discuss the advantages and limitations of serological surveys as a mean to disclose pathogen circulation in wildlife at population level. 

Whatever the scope of disease monitoring, and especially in a conservation context, opportunistic sampling is sometimes the only strategy available to obtain information on the health status of wild animal populations. Rohner et al. [7] and Fusillo et al. [8] apply standardised post-mortem protocols on opportunistically collected carcasses to investigate causes of mortality in Eurasian otter (*Lutra lutra*) populations in the north of Germany and the south of Italy, respectively. The Eurasian otter is an elusive mammal classified as near threatened globally (IUCN Red List), and these contributions therefore offer some useful insights to better target conservation efforts. Both groups detected an increased mortality in autumn-winter, an age distribution skewed towards subadults, and identified vehicle collisions as the main cause of death for otters. Another contribution in the context of animal conservation describes the opportunistic assessment of squirrelpox disease in Eurasian red squirrels (*Sciurus vulgaris*) in Wales (UK). Shuttleworth et al. [9] relied on a combination of capture-mark-recapture data, camera trapping, and retrieval of carcasses by citizens and operators (aided also by conservation dogs) to monitor disease outbreaks in a population of the native rodent sympatric with invasive Eastern grey squirrels (*S. carolinensis*), a known reservoir for the virus. 

The opportunistic collection of carcasses of wild boars (*Sus scrofa*) and macaques (*Macaca fascicularis*) was employed also by Lekko et al. [10] to investigate the circulation of *Mycobacterium tuberculosis* complex (MTBC) and *Mycobacterium avium* complex (MAC) at the wildlife–livestock–human interface in Malaysia. In light of their findings, the authors discuss best practices for MTBC and MAC detection and surveillance in wildlife and for limiting disease spread and transmission to livestock and humans. 

This Special Issue also offers some valuable insights regarding overlooked and underreported causes of disease in wildlife. Following a clinical case in a military macaw (*Ara militaris*) housed in a Portuguese zoo, Marques et al. [11] report on the isolation of the opportunistic fungus *Exophiala* spp. from the upper respiratory tract of twelve different parrot species. Although they sampled captive individuals, and no clear pathogenic role of this microorganism emerged, their report provides additional insight into the microbiome of tropical birds, a still underexplored topic. Ash and Patterson [12] aimed to fill the knowledge gap on cyanotoxin poisoning in terrestrial wildlife, a phenomenon which is bound to increase in frequency due to climate warming and water eutrophication. The authors review the available evidence regarding mortality and morbidity in wildlife populations associated with harmful cyanobacteria blooms in freshwater, recommend methods for their correct investigation and reporting, and discuss potential mitigation measures.

Finally, Schilling et al. [13] reviewed the literature on non-invasive methods in wildlife disease and health research. In their contribution, they analyse the publication trends on the topic, revealing the growing interest of researchers in employing non-invasive methods, then offer a comprehensive review of the different types of samples that can be collected non-invasively from wildlife, listing their potential uses and methods for collection and analysis.

We would like to thank all the colleagues that contributed to this Special Issue, which we believe may offer some useful insights to researchers and professionals dealing with wildlife diseases and health. We hope that the collated experiences will help with the design of future projects and spark conversations between different groups already working or only just setting out in the field, with the ultimate goal of protecting wildlife health and with it environmental, livestock and human health in a One Health framework.
